# Evolutionary and Experimental Assessment of Novel Markers for Detection of *Xanthomonas euvesicatoria* in Plant Samples

**DOI:** 10.1371/journal.pone.0037836

**Published:** 2012-05-24

**Authors:** Pedro Albuquerque, Cristina M. R. Caridade, Arlete S. Rodrigues, Andre R. S. Marcal, Joana Cruz, Leonor Cruz, Catarina L. Santos, Marta V. Mendes, Fernando Tavares

**Affiliations:** 1 IBMC-Instituto de Biologia Molecular e Celular, Universidade do Porto, Porto, Portugal; 2 FCUP-Faculdade de Ciências, Departamento de Biologia, Universidade do Porto, Porto, Portugal; 3 CICGE-Centro de Investigação em Ciências Geo-Espaciais, Faculdade de Ciências, Universidade do Porto, Porto, Portugal; 4 ISEC-Instituto Superior de Engenharia de Coimbra, Coimbra, Portugal; 5 CMUP-Centro de Matemática da Universidade do Porto, Faculdade de Ciências, Universidade do Porto, Porto, Portugal; 6 INRB-Instituto Nacional de Recursos Biológicos, Unidade de Investigação de Protecção de Plantas, Tapada da Ajuda, Lisboa, Portugal; 7 CIBIO-Centro de Investigação em Biodiversidade e Recursos Genéticos, Universidade do Porto, Campus Agrário de Vairão, Vairão, Portugal; Naval Research Laboratory, United States of America

## Abstract

**Background:**

Bacterial spot-causing xanthomonads (BSX) are quarantine phytopathogenic bacteria responsible for heavy losses in tomato and pepper production. Despite the research on improved plant spraying methods and resistant cultivars, the use of healthy plant material is still considered as the most effective bacterial spot control measure. Therefore, rapid and efficient detection methods are crucial for an early detection of these phytopathogens.

**Methodology:**

In this work, we selected and validated novel DNA markers for reliable detection of the BSX *Xanthomonas euvesicatoria* (*Xeu*). *Xeu-*specific DNA regions were selected using two online applications, CUPID and Insignia. Furthermore, to facilitate the selection of putative DNA markers, a customized C program was designed to retrieve the regions outputted by both databases. The *in silico* validation was further extended in order to provide an insight on the origin of these *Xeu*-specific regions by assessing chromosomal location, GC content, codon usage and synteny analyses. Primer-pairs were designed for amplification of those regions and the PCR validation assays showed that most primers allowed for positive amplification with different *Xeu* strains. The obtained amplicons were labeled and used as probes in dot blot assays, which allowed testing the probes against a collection of 12 non-BSX *Xanthomonas* and 23 other phytopathogenic bacteria. These assays confirmed the specificity of the selected DNA markers. Finally, we designed and tested a duplex PCR assay and an inverted dot blot platform for culture-independent detection of *Xeu* in infected plants.

**Significance:**

This study details a selection strategy able to provide a large number of *Xeu*-specific DNA markers. As demonstrated, the selected markers can detect *Xeu* in infected plants both by PCR and by hybridization-based assays coupled with automatic data analysis. Furthermore, this work is a contribution to implement more efficient DNA-based methods of bacterial diagnostics.

## Introduction

Every year, heavy yield losses in the agricultural production of many countries are attributed to phytopathogenic bacteria. Moreover, with the globalization of trade, the worldwide import and export of food crops facilitates the risk of the rapid spreading of such bacteria. Therefore, efficient and rapid quarantine procedures are required, not only to prevent pathogen spreading, but also to manage the already infected areas [1]. The genus *Xanthomonas* comprises many phytopathogenic species [2] and a total of thirteen genus members are considered as quarantine organisms by EPPO (European and Mediterranean Plant Protection Organization). Bacterial spot-causing xanthomonads (BSX) are amongst EPPO's A2 list of quarantine organisms (“*Xanthomonas axonopodis* pv. *vesicatoria*” and “*Xanthomonas vesicatoria*”) and are suspected to occur all over the Mediterranean area [3].

BSX were initially classified as a single taxon: *Xanthomonas campestris* pv. *vesicatoria* (*Xcv*), which was believed to be an homogeneous group. However, polyphasic approaches clearly showed two different lineages within *Xcv*: group A (*X. axonopodis* pv. *vesicatoria*) and group B strains (*X. vesicatoria*) [4], [5], [6]. Later, with the isolation of novel BSX that differed from group A and B strains coupled with further DNA-DNA hybridization studies, four distinct groups of BSX were considered and a new nomenclature was proposed: group A strains as *X. euvesicatoria*, group B as *X. vesicatoria*, group C as *X. perforans*, and group D as *X. gardneri* [7], [8], [9]. In this work, BSX strains are referred according to this quadripartite nomenclature. Multi Locus Sequences Analysis and *gyrB*-based phylogeny placed *X. euvesicatoria* and *X. perforans* in a single clade, while *X. gardneri* were considered close to *X. hortorum* and *X. cynarae*. In turn, *X. vesicatoria* were consistently positioned in a distinct clade [10], [11].

To date, the use of healthy greenhouse seedlings and seed lots is still considered as the most effective bacterial spot control measure [3], [12], requiring the development of effective BSX detection methods. Nevertheless, the disease control is still frequently reliant on the use of standard copper sprays, however, its phytotoxic effects and the resistance displayed by some strains led to the evaluation of alternative spraying methods [13], [14], [15]. Biological control [16], [17], [18], [19] and the use of resistant cultivars [20], [21], [22] have recently gained an increasing importance as means of disease control. Despite the fact that culture-based methods remain the gold-standard for bacteria detection in official laboratories [3], DNA-based methods of detection are now acknowledged as unquestionable alternatives [23], [24], [25], [26], and a large number of approaches have already been validated, being currently applied in routine surveys.

In order to become standard tools for detection of BSX, i.e. officially recognized by the phytosanitary services, DNA-based methods must be highly specific for the target pathogen and must provide reliable detection results, namely by applying several DNA markers simultaneously. Furthermore, they are required to be rapid and undemanding to perform, ideally allowing the direct detection in plant material against a complex microbial background. The efficiency of these molecular detection methods is mainly dependent on two factors: the selection of target-specific DNA regions (DNA signatures) and the use of appropriate techniques for the detection of those DNA signatures. While new and improved techniques, with the potential to be applied in bacterial diagnostics, are continuously reported in the literature, the proficient selection of target-specific DNA regions is still hampered by the lack of efficient signature selection pipelines [27]. For the DNA-based detection of BSX, a few detection markers have already been suggested, including genes related to copper resistance [28], genes required for expression of lipopolysaccharide epitopes [29], the *hrp* genes [30], [31], [32], an *rhs* family gene [33] or a type IV fimbrial-subunit gene (*fimA*) [34]. Non-characterized genomic regions, discovered via subtractive hybridization [35] or fingerprinting methods [36], have been described as well. The analysis of restriction patterns and other DNA fingerprinting methods for identification of BSX isolates has also been explored [37], [38], [39], [40] and, in some instances, these procedures are necessary for the confirmation of the detection results [29], [32]. However, further research is still needed in order to improve both the specificity and reliability of BSX detection methods.

The continuously increasing amount of sequence data in publicly available databases, and the current comparative genomics tools, allow to select a large number of potential DNA signatures and to perform meaningful *in silico* specificity tests, which make possible to focus the laborious and time-consuming “wet lab” validation assays in pre-selected and optimized markers [27]. CUPID [41] and Insignia [42] are online-based resourceful bioinformatics applications made for this purpose with user-friendly interfaces and freely available. CUPID is a database of taxa-specific proteins calculated via an automated BLAST-reverse BLAST analysis. This sequential BLAST analysis of all proteins identified in a given proteome outputs the proteins that are specific to different taxonomic levels: strain, species and genus. Insignia is based on a DNA signature discovery pipeline that calculates target-specific DNA regions, according to a series of user-defined experimental constraints. Like CUPID, this online database allows to easily retrieve specific regions for different taxonomic levels.

In this work, CUPID and Insignia were used to select novel DNA signatures specific for the fully sequenced BSX *Xanthomonas euvesicatoria* str. 85-10 (*Xeu* 85-10) [43]. The selected signatures were obtained by overlapping both databases outputs with a customized C program. Additionally, comparative genomics and phylogenomic-related tools were used to assess the evolutionary history of these highly specific regions. This information provided some insights about the evolutionary origin and the stability of the regions selected as putative DNA markers. These regions were then validated using both PCR and hybridization-based approaches. The most promising *Xeu*-specific markers were used to detect the pathogen in infected plant samples using both a duplex PCR, for time-efficient and easy detection, as well as an inverted dot blot platform, using six markers simultaneously. Furthermore, software previously developed by us [44], [45], was used for the automatic processing of dot blot results, both at the validation and detection stages, to uniformly analyze the obtained hybridization data.

## Materials and Methods

### *In silico* selection of *X. euvesicatoria* specific DNA regions

For the selection of *Xanthomonas euvesicatoria* specific DNA regions two online-based databases were used: CUPID (http://pir.georgetown.edu/cupid) and Insignia (http://insignia.cbcb.umd.edu/). CUPID was applied to list all the proteins that were calculated as specific for the sequenced strain *Xeu* 85-10. Afterwards, this list was cross-analyzed with Uniprot and NCBI databases to link CUPID's outputted protein accession numbers to their corresponding gene name and location in genome (genome coordinates). Insignia was used to calculate 20 mer DNA signatures specific for strain *Xeu* 85-10. The output was then filtered for signature chains (consecutive 20 mer signatures) higher than 100 bp and 260 bp and the non-chromosomal data was filtered out from both data sets. The outputs of both databases were analyzed with a custom-made C program, available upon request, that allowed the determination of the overlaps between the genome coordinates of CUPID's corresponding nucleotide sequences and the coordinates of both sets of Insignia's outputted signatures. The confirmatory *in silico* specificity tests were performed using the BLAST (blastn) utility [46] and ten regions were selected for experimental validation (Table 1).

**Table 1:** Primer-pairs and best BLAST hits for the selected markers.

DNA Marker	Target gene	Primer	Sequence (5′-3′)	Amplicon Lengh (bp)	Amplicon best BLAST hit (E value/query coverage)
**XV4**	XCV0215	XV4F	ATCAATGAGCCTTGGGATGTGACGA	230	*Corallococcus coralloides* DSM 2259
		XV4R	GCATAGGTCAGGGCTTGCTTTAGCG		3.7/13%
**XV5**	XCV0217	XV5F	GCCTAAGAATGCGGAGCCTTGGCT	210	*Neospora caninum* Liverpool
		XV5R	ATCTTCGGAGGCGTGTACGGCGTA		3.3/10%
**XV6**	XCV3374	XV6F	AATGTGATCTTTTTGACGAGCGCA	169	*Stenotrophomonas maltophilia* K279a
		XV6R	GCAACCTCGTCTGTTTCATTCTCAT		0.017/21%
**XV7**	XCV3818	XV7F	CATTTCCATCACGCGTCATGCCG	179	*Xanthomonas axonopodis* pv. *citrumelo* F1
		XV7R	TGTTGCTCGGAATCGGTGGACCACC		2e-85/100%
**XV8**	XCV3902	XV8F	TGTCTCAAGCCGCGCTTAAC	123	*Pantoea ananatis* PA13
		XV8R	AACCGAAGAACAGGAACGATCTC		0.003/50%
**XV10**	XCV0217	XV10F	GCGTTGGCACAATGTCGACC	805	*Bradyrhizobium japonicum* USDA 110
		XV10R	TTCGTCTAGCTCTCCACGGACCTG		0.081/4%
**XV11**	XCV0655	XV11F	GCGACTGCGCTGGTATGAGCTCTA	631	*Xanthomonas axonopodis* pv. *citrumelo* F1
		XV11R	TGGCGTGTAGACACCCACTGTCGAG		0.0/100%
**XV12**	XCV1116	XV12F	GGAGCCGTCTGCTGGTAAGCTGAT	638	*Propionibacterium freudenreichii* CIRM-BIA1
		XV12R	GCTGTATCAAACGAGATCCGCTG		0.26/10%
**XV13**	XCV1303	XV13F	TCACATTCTCATCACAGGACCCTG	836	*Xanthomonas albilineans* GPE PC73
		XV13R	ATGTCCTCACGAGTGCCGGA		8e-41/26%
**XV14**	XCV1853	XV14F	TGGTTCACGTCATCGTTGTCGGA	713	*Xanthomonas albilineans* GPE PC73
		XV14R	TAGAGCTCGCTCAAAGCCCTTCGG		0.007/9%

### *In silico* analysis of selected regions

Several *in silico* analyses were carried out in order to gain an insight on the evolutionary origin of the *Xeu*-specific regions selected. The circular chromosome map was visualized using Geneious Pro [47], and the position of each selected DNA marker was pinpointed along with all the phage related ORFs, IS elements, tRNAs, recombinases, integrases and transposases annotated in the genome of *Xeu* 85-10. The Codon Adaptation Index (CAI), the expected CAI (eCAI) and GC percentages were calculated using the CAIcal server [48]. For comparison purposes, these parameters were also considered for four housekeeping genes (*atpD*, *dnaK*, *efP2* and *gyrB*).

Synteny analyses were carried out using both SynMap, for generating whole genome syntenic dotplots, and GEvo, for high-resolution analysis (40 Kb intervals) of selected genomic regions, two applications from the CoGe platform of comparative genomics [49], [50].

### Bacterial strains and culture conditions

The bacterial strains used in this study are listed in Table 2. All *Xanthomonas* and *Stenotrophomonas maltophilia* strains were cultured in YGC medium containing glucose (10 g.L^−1^), yeast extract (5 g.L^−1^), CaCO_3_ (30 g.L^−1^) and agar (15 g.L^−1^) at 28°C; except for *Xanthomonas fragariae*, which was cultured in YPGA medium containing yeast extract (5 g.L^−1^), bacto peptone (5 g.L^−1^), glucose (10 g.L^−1^) and agar (15 g.L^−1^) at 20°C. All the non-*Xanthomonas* strains were cultured in Nutrient Agar with beef extract (1 g.L^−1^), yeast extract (2 g.L^−1^), peptone (5 g.L^−1^), NaCl (5 g.L^−1^), KH_2_PO_4_ (0.45 g.L^−1^), Na_2_HPO_4_ ·12H_2_O (2.39 g.L^−1^) and agar (15 g.L^−1^), except for *Xylella fastidiosa* which was cultured in BCYE media [51]. *Escherichia coli* were cultured on Luria-Bertani medium at 37°C. Standard *E. coli* manipulation and *in vitro* DNA manipulations were carried out as described by Sambrook and Russell [52].

**Table 2:** List of bacterial strains used in this study.

Strain (acronym)	Source*	Geographic origin
*Xanthomonas euvesicatoria* (*Xeu*)^a^	LMG 667; LMG 668; LMG 904;	NM; Cook Island; NM
	LMG 905; LMG 906; LMG 909;	NM; NM; Cote D'ivoire
	LMG 910; LMG 913; LMG 914;	Morocco; Senegal; Senegal
	LMG 922; LMG 926; LMG 929;	USA, Hungary; USA
	LMG 930; LMG 931; LMG 932;	USA; USA; Brazil
	LMG 933; CPBF 404 (985-B7);	Brazil; Spain;
	CPBF 490 (isolate); LMG 907	Spain; India
*Xanthomonas vesicatoria* (*Xv*)^a^	LMG 911^c^, LMG 917,	New Zealand; New Zealand;
	LMG 919, LMG 920, LMG 923	Zimbabwe; Italy; Hungary
*Xanthomonas gardneri* (*Xg*)^a^	LMG 962^c^, NCPPB 4323,	Yugoslavia; Costa Rica;
	NCPPB 4324	Costa Rica
*Xanthomonas perforans* (*Xp*)^a^	NCPPB 4321^c^, NCPPB 4322	USA; USA
*Xanthomonas arboricola* pv. *celebensis* (*Xac*)	LMG 677^b^	New Zealand
*Xanthomonas arboricola* pv. *corylina* (*Xaco*)	LMG 689^b^	USA
*Xanthomonas arboricola* pv. *juglandis* (*Xaj*)	LMG 747^b^	New Zealand
*Xanthomonas arboricola* pv. *pruni* (*Xap*)	LMG 852^b^	New Zealand
*Xanthomonas axonopodis* pv. *citri* (*Xaci*)	LMG 9322^c^	USA
*Xanthomonas axonopodis* pv. *dieffenbachiae* (*Xad*)	LMG 695^b^	Brazil
*Xanthomonas axonopodis* pv. *phaseoli* (*Xaph*)	LMG 7455	USA
*Xanthomonas campestris* pv. *campestris* (*Xcc*)	LMG 568^b^	United Kingdom
*Xanthomonas fragariae* (*Xf*)	LMG 708	USA
*Xanthomonas oryzae* pv. *oryzae* (*Xoo*)	LMG 5047^b^	India
*Xanthomonas oryzae* pv. *oryzicola* (*Xooa*)	LMG 797^b^	Malaysia
*Xanthomonas translucens* pv. *translucens* (*Xtt*)	LMG 876^b^	USA
*Clavibacter michiganensis* subsp. *michiganensis* (*Cmm*)	LMG 7333^c^	Hungary
*Erwinia amylovora* (*Ea*)	LMG 2024^c^	United Kingdom
*Pectobacterium atrosepticum* (*Pa*)	LMG 2386^c^	United Kingdom
*Pectobacterium carotovorum* subsp. *carotovorum* (*Pcc*)	LMG 2404^c^	Denmark
*Pectobacterium chrysanthemi* (*Pch*)	LMG 2804^c^	USA
*Pseudomonas fluorescens* (*Pf*)	Pf0-1	USA
*Pseudomonas putida* (*Pp*)	KT 2440	Japan
*Pseudomonas savastanoi* pv. *glycinea* (*Psvg*)	LMG 5066	New Zealand
*Pseudomonas savastanoi* pv. *phaseolicola* (*Psvp*)	LMG 2245	Canada
*Pseudomonas syringae* pv. *helianthi* (*Psh*)	LMG 5067^b^	Mexico
*Pseudomonas syringae* pv. *maculicola* (*Psm*)	LMG 5071^b^	New Zealand
*Pseudomonas syringae* pv. *oryzae* (*Pso*)	LMG 10912^b^	Japan
*Pseudomonas syringae* pv. *syringae* (*Pss*)	DSM 10604^b^	United Kingdom
*Pseudomonas syringae* pv. *tabaci* (*Pstb*)	LMG 5393^b^	Hungary
*Pseudomonas syringae* pv. *tomato* (*Pst*)	DC 3000	United Kingdom
*Ralstonia picketii* (*Rp*)	LMG 5942^c^	USA
*Ralstonia solanacearum* (*Rs*)	LMG 2299^c^; LMG 2302; LMG 2306;	USA; Costa Rica; Portugal;
	LMG 17138; LMG 17140	Brazil; Sweden
*Stenotrophomonas maltophilia* (*Sm*)	LMG 958^c^	USA
*Xylella fastidiosa* (*Xllf*)	LMG 17159^c^	USA

* LMG-Belgian Co-Ordinated collections of micro-organisms, Gent, Belgium; CPBF-Colecção Portuguesa de Bactérias Fitopatogénicas, Lisboa, Portugal; NCPPB-National Collection of Plant Pathogenic Bacteria, York, United Kingdom.

a - Bacterial spot-causing xanthomonads (BSX);

b - Pathovar reference strain;

c - Type strain; NM- Not mentioned.

### PCR validation of the selected markers

DNA was extracted from axenic bacterial cultures using the EZNA Bacterial DNA Purification Kit (Omega Bio-Tek, Norcross, GA), following the manufacturer's instructions, and quantified using a NanoDrop spectrophotometer (Thermo Scientific, Wilmington, DE).

Primer pairs were designed using the Vector NTI 10 software (Invitrogen, Carlsbad, CA), with a calculated annealing temperature of approximately 60°C (Table 1).

The PCR mastermix contained 1× Reaction Buffer IV (ABgene, Epsom, UK), 0.2 mM of each dNTP (Fermentas, Ontario, Canada), 1.5 mM of MgCl_2_, 0.2 µM of each primer and 1 U of Simple Red DNA Polymerase (ABgene). 25 ng of pure genomic DNA were used as template. The PCR conditions were as follows: an initial denaturation step of 5 min at 95°C, followed by 35 cycles of 30 s at 95°C, 30 s at 57°C, 59°C or 61°C and 30 s at 72°C with a final extension step of 10 min at 72°C. Amplicons were extracted and purified from agarose gels stained with ethidium bromide (Bio-Rad, Hercules, CA), using the GFX PCR and Gel Band Purification kit (GE Healthcare, Buckinghamshire, UK). Purified amplicons were cloned in pGEM-T easy vector (Promega, Madison, WI), according to the manufacturer's instructions, and their identity was confirmed by sequencing (STAB Genomica, Portugal).

The duplex PCR was carried out as mentioned above, using 1.5 U of Simple Red DNA Polymerase (ABgene) and with the PCR conditions altered to: 35 cycles of 30 s at 95°C, 30 s at 61°C and 45 s at 72°C.

### Genomic diversity of selected BSX strains

To determine if the BSX strains selected for specificity validation were representative of the group's genomic diversity, a Neighbor-Joining Tree was constructed using MEGA 5 [53]. The tree was based on the concatenated sequences of genes *atpD*, *dnaK*, *efp* and *gyrB* of several BSX and other *Xanthomonas*. The same software was used to calculate an appropriate evolutionary model and TN93+G+I was selected, which corresponds to the Tamura-Nei model with a rate variation among sites modulated by the gamma parameter and considering a proportion of invariable sites. Tree consistency was assured by 1000 bootstrap replicates.

### Dot blot specificity assays and automatic analysis of hybridization data

For Dot blot assays, 100 ng of heat-denatured DNA from pure bacterial cultures were spotted into a nylon membrane using a Bio-Dot apparatus (Bio-Rad, Hercules, CA). DNA probes were obtained from purified PCR amplicons labeled with digoxigenin, using the DIG-High Prime labeling kit (Roche, Basel, Switzerland) and following the manufacturer's instructions. Hybridization was carried out overnight at 68°C, with a final probe concentration of 100 ng.ml^−1^. Washing and detection steps were conducted according to the manufacturer's instructions. DIG-labeled nucleic acids were detected by chemiluminescence and the dot blot images were acquired with a Molecular Imager ChemiDoc system (Bio-Rad), adjusting the exposure time so that all dots were below pixel saturation.

The analysis of hybridization data was done using an algorithm developed to automatically process the dot blot images. Besides adjusting each image to a user-defined grid, this software outputs the probability values of each dot being a positive signal, using as references the positive and negative controls present in each membrane [44], [45].

### PCR and hybridization-based detection of BSX in infected plant material

For validation of the selected markers and detection techniques using plant material, seeds of *Capsicum annuum* and *Solanum lycopersicum* were grown in a plant growth chamber (24°C, 16 h/8 h photoperiod, 3500 Lux of light intensity, and 50% of relative humidity) until the fourth true leaf stage. The leaves were sprayed with approximately 10^6^ cells per mL of selected BSX and the infected leaves were collected on the first and second weeks after infection. For negative controls, plants were also infected with *Pseudomonas syringae* pv. *tomato* (*Pst* DC3000).

Leaf samples were macerated with a micropipette tip in a 50 ml conical tube containing 10 mL of sterile distilled water. Two milliliters of the supernatant were recovered to a microcentrifuge tube and centrifuged at 10.000 g for 2 min. The supernatant was discarded and the pellet ressuspended in 200 µL of sterile distilled water, as a crude bacterial suspension.

For the Duplex PCR assays, using primers XV7 F/R and XV11 F/R, 10 µL of each bacterial suspension sample were used directly for PCR amplification. To promote cell lysis an initial denaturation step of 10 min at 95°C was added.

For inverted dot blot assays, 100 ng of each purified PCR product, corresponding to each marker and to the 16 S rRNA gene, were spotted on a nylon membrane. The amplicons were obtained using DNA template from strain *Xeu* 905 using the primers (Table 1) and PCR conditions as described above. For the amplification of the 16 S rRNA gene, used as a positive control, the primer pair 357f/519r was used [54]. For each infected plant sample, and in order to improve the detection resolution, a PCR enrichment step was carried out, using the seven primer-pairs simultaneously. The obtained amplicons were purified and labeled with Digoxigenin as described before. Hybridization, washing and detection conditions were the same as mentioned above.

### Nucleotide sequences accession numbers

DNA sequences were deposited in the NCBI database with accession numbers HQ316640 to HQ316699.

## Results

### Selection of *Xeu* specific DNA markers

The selection of *Xeu* putative DNA markers was carried out using CUPID, Insignia and a C program, designed to overlap the outputs of the two databases and produce a single set of results. Taking into account that the distribution of plasmids across different *Xeu* strains is highly variable and dynamic [55], and given that plasmid-based markers could easily lead to false-negative results, the selection of DNA signatures only included chromosomal data.

CUPID was used to select *Xeu* unique proteins, which generated 195 unique entries, of which 149 were encoded by chromosomal genes. Insignia was used to output *Xeu*-specific 20 mer signatures, with a total of 15533 signatures obtained. In order to allow the optimization of a duplex PCR, the *in silico* analysis aimed at two sets of DNA markers of different size: one set of small molecular markers (∼200 bp) and one set of larger markers (∼700 bp). For the smaller set, Insignia's output was filtered for a signature chain length (consecutive 20 mer signatures) higher than 100 bp. The use of larger signature chains, apart from allowing to narrow down the number of obtained signatures, is also acknowledged to improve the specificity of the outputted DNA regions [56]. A total of 3768 signatures were outputted, 3071 in the chromosome. From these, 173 signatures were shown to overlap with CUPID's output using the custom-made C program, which corresponded to 104 different genes spread throughout the whole chromosome. Five regions, whose specificity was sustained by BLAST, were randomly selected for further analysis and named XV4, XV5, XV6, XV7 and XV8. For the larger DNA markers set, Insignia's signature chain length was increased to 260 bp and 398 signatures were outputted, with 295 of them present in the chromosome. In this case, CUPID and Insignia overlapped in 19 regions, which corresponded to 16 different genes. A BLAST analysis revealed that five of the overlapped regions were not completely specific for *Xeu*. From the remaining regions, five were selected and identified as XV10, XV11, XV12, XV13 and XV14 (Table 1). Overall, from the ten selected regions, only XV7 and XV11 presented significant BLAST hits with *Xanthomonas axonopodis* pv. *citrumelo* F1, a non-target bacteria recently sequenced [57]. It should be noted that the gene tagged as XCV0217, obtained in the two data sets, was used to design the low size XV5 (210 bp) and the large size XV10 (805 bp) markers.

### Comparative genomic analysis of *Xeu*-specific markers

To gain further insight concerning the uniqueness of these *Xeu*-specific genomic regions, which is an important feature for a secure *in silico* selection of *Xeu* markers for detection, a thorough comparative genomic analysis was carried out.

**Figure 1:**
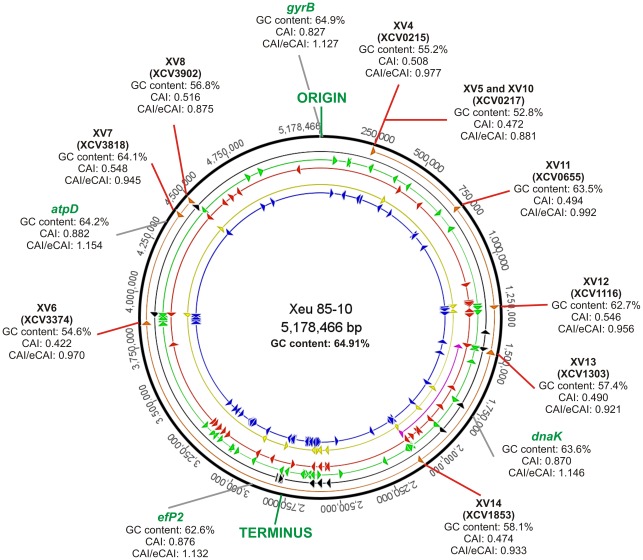
Genome map of *X. euvesicatoria* str. 85-10. Circles, from the outside in, show: genome coordinates (bp), selected DNA markers (orange), phage related ORFs (black), IS elements (green), tRNAs (red), recombinases (purple), integrases (yellow) and transposases (blue). The GC content, Codon Adaptation Index (CAI) and normalized CAI (CAI/eCAI) values are shown for each marker and for four housekeeping genes.

Interestingly, the chromosomal location of the *Xeu*-specific markers (Fig. 1) shows that, with exception for markers XV11 and XV7, the markers are present in the vicinity of mobilization-related features, namely phage related ORFs, IS elements, tRNAs, recombinases, integrases and transposases, which suggest high genomic plasticity. The hypothesis that most markers were likely result of horizontal gene transfer is further supported by the GC content, the Codon Adaptation Index (CAI) and their expected values (e-CAI), and by comparative syntenic maps. In fact, with the exception for markers XV7, XV11 and XV12, the GC content of the markers is clearly below the reported value of 64.91% GC for *Xeu* 85-10 (Fig. 1). Concerning the CAI and their normalized values (CAI/e-CAI), the numbers obtained for the markers are consistently below the values obtained for four housekeeping genes used as reference (*dnaK*, *efP*2, *atpD* and *gyrB*), which signify a divergence in codon usage [58].

The synteny analysis performed allowed whole genome comparisons of *Xeu* against *X. axonopodis* pv. *citri* str. 306 (*Xaci* 306), *X. campestris* pv. *campestris* ATCC 33913 (*Xcc* 33913) and *Xanthomonas oryzae* pv. *oryzae* MAFF 311018 (*Xoo* 311018). (See Fig. S1, Fig. S2 and Fig. S3). The obtained syntenic dotplots, further highlighted by the high resolution analysis, showed that most markers were placed in small discontinuities in the syntenic lines, which are characteristic of genomic rearrangements. A more detailed comparison with *Xaci* 306 (Fig. S1), with 40 Kb intervals around each marker, confirmed that all markers, with exception of XV6, XV7 and XV11, were located between flanking syntenic regions, suggesting insertion events. This pattern is further corroborated with *Xcc* 33913 (Fig. S2). Interestingly, markers XV7 and XV11 are again contained within reasonably similar genomic regions, although the analysis suggests an inversion event in the synton surrounding XV11. As expected [43], the syntenic analysis with *Xoo* 311018 (Fig. S3) illustrated a completely different genomic structure. Nevertheless, markers XV7 and XV11 are close to high similarity regions.

### PCR and amplicon sequences analysis

Ten primer pairs (Table 1) were designed for amplification of the selected regions with a calculated annealing temperature of around 60°C, in order to achieve standardized PCR conditions.

**Figure 2:**
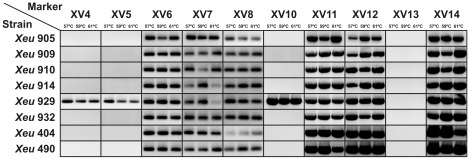
PCR validation. The selected primer-pairs were tested for efficiency using eight different *Xeu* strains. For each assay, three different annealing temperatures were tested: 57°C, 59°C and 61°C.

The primers specificity was assessed with eight *Xeu* strains representative of a broad range of geographic origins (Fig. 2, Table 2). The results showed that markers XV6, XV7, XV8, XV11, XV12 and XV14 provided positive amplification with all the tested strains whatever the annealing temperature, contrary to markers XV4, XV5 and XV10 which were amplified only with strain *Xeu* 929, and marker XV13 that was not amplified whatever the strain and the PCR conditions. The amplicons corresponding to the markers shown to be present in all the *Xeu* tested strains were sequenced to confirm their identity, and to infer the intraspecific variability, which was shown to be low as demonstrated by minor nucleotide differences between the *Xeu* strains analyzed (see Table S1).

### Dot blot specificity analysis

**Figure 3:**
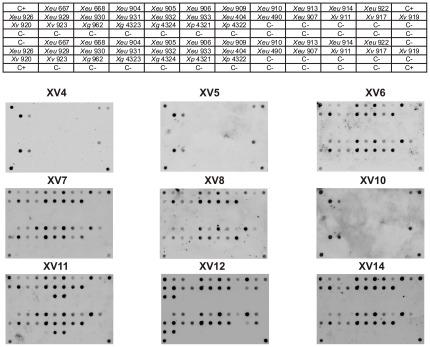
Dot blot validation of selected probes. Nine probes were evaluated with total DNA from a collection of BSX, consisting of 19 *Xeu*, five *Xv*, three *Xg* and two *Xp* strains. Probability values, obtained with a customized MATLAB algorithm for the automatic data analysis, are detailed in Table 3.

**Figure 4:**
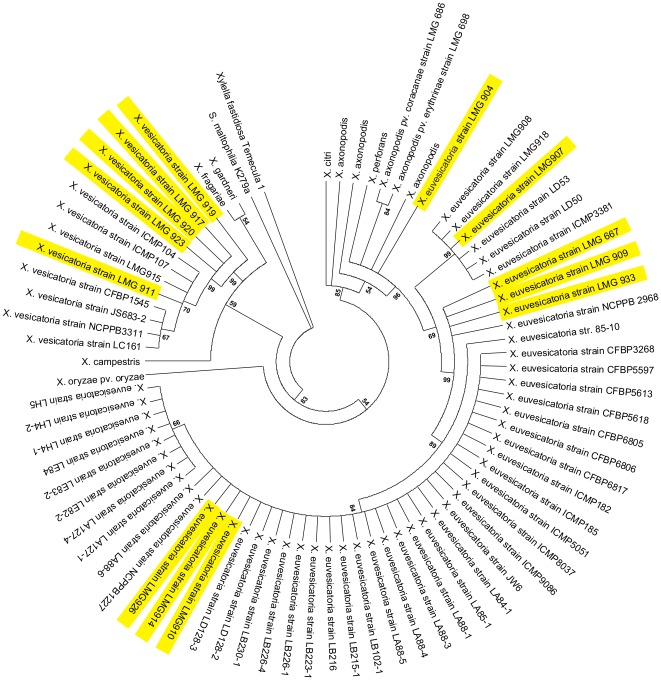
Neighbor-Joining Tree based on the concatenated sequences of four housekeeping genes of several *Xanthomonas*. The sequences of the housekeeping genes *atpD*, *dnaK*, *efp* and *gyrB* were concatenated and used to infer the MLST profile of *X. euvesicatoria* and *X. vesicatoria* strains used in this study, which are highlighted in yellow. The Neighbor-Joining tree was derived from the TN93+G+I model and a bootstrap analysis of 1000 replicates.

Specificity validation of the markers using a dot blot hybridization procedure was extended to a larger set of BSX including 19 *Xeu* strains, five *Xanthomonas vesicatoria* (*Xv*), three *Xanthomonas gardneri* (*Xg*), and two *Xanthomonas perforans* (*Xp*) (Fig. 3). 12 non-BSX xanthomonads and 23 non-*Xanthomonas* were used to assess unspecific binding (See Fig. S4). Furthermore, the BSX strains used for this validation, in addition to their diverse geographic origin (Table 2), also corresponded to well distinct lineages. In fact, as inferred by the MLST profile obtained by the Neighbor-Joining analyses of the concatenated sequences of four housekeeping genes (*atpD*, *dnaK*, *efp* and *gyrB*) (Fig. 4), the *Xeu* and *Xv* strains used in this study provide a very good coverage of the observed phylogenetic clusters.

For the dot blot specificity assays, the probes corresponded to the digoxigenin-labeled PCR products for markers XV6, XV7, XV8, XV11, XV12 and XV14 obtained with strain *Xeu* 905. For markers XV4, XV5 and XV10, the probes corresponded to the amplicons obtained with strain *Xeu* 929, since it was the only strain that provided amplification for these markers. In order to ensure a reliable assessment of the hybridization data and to overcome the biased human interpretation of dot blot images, we used a ChemiDoc system that allowed the acquisition of images just below saturation of any pixel, and an algorithm to computerize the dot blot images, as previously described [59]. Briefly, this application determines the probability value of a positive signal for each dot, allowing the comparison between dots in different positions in the membrane and from independent hybridization experiments (Table 3).

**Table 3:** Outputted probability values concerning the dot blot validation assays with a collection of BSX strains.

Strain	Calculated ON probability								
	XV4	XV5	XV6	XV7	XV8	XV10	XV11	XV12	XV14
*Xeu* LMG 667	0±0	0±0	**0.71±0.27**	**0.91±0.05**	**0.77±0.27**	0±0	**0.89±0.05**	**0.97±0.03**	**0.83±0.12**
*Xeu* LMG 668	0.01±0.01	0±0	**0.85±0.15**	**0.93±0.06**	**0.82±0.15**	0.12±0.17	**0.9±0.1**	**0.97±0.03**	**0.93±0.05**
*Xeu* LMG 904	0.01±0.01	0±0	0.16±0.26	**1±0.01**	0.03±0.03	0.2±0.28	**1±0**	0.01±0.01	0.01±0.01
*Xeu* LMG 905	0.01±0	0±0	**1±0**	**1±0**	**1±0**	0.14±0.18	**1±0**	**1±0.01**	**1±0**
*Xeu* LMG 906	0±0	0±0	**0.96±0.05**	**1±0.01**	**0.9±0.06**	0±0	**1±0**	**0.83±0.24**	**0.98±0.02**
*Xeu* LMG 909	0±0	0±0	**1±0**	**1±0**	**1±0**	0±0	**1±0**	**0.99±0.02**	**1±0**
*Xeu* LMG 910	0.01±0.01	0.01±0.01	**0.99±0.01**	**0.99±0.02**	**0.96±0.03**	0.06±0.08	**0.97±0.05**	**0.98±0.02**	**0.98±0.04**
*Xeu* LMG 913	0±0	0.03±0.04	**0.61±0.4**	**0.61±0.28**	0.33±0.17	0±0	**0.73±0.27**	**0.67±0.33**	**0.71±0.24**
*Xeu* LMG 914	0±0	0.04±0.06	**1±0**	**1±0**	**1±0**	0±0	**1±0**	**1±0**	**1±0**
*Xeu* LMG 922	**0.93±0.04**	**1±0.01**	**0.82±0.16**	**0.93±0.03**	**0.64±0.18**	**1±0**	**0.95±0.04**	**0.9±0.1**	**0.86±0.23**
*Xeu* LMG 926	0.02±0.01	0±0	**0.99±0.01**	**0.99±0.01**	**0.74±0.31**	0.04±0.05	**0.99±0.02**	**1±0**	**1±0.01**
*Xeu* LMG 929	**1±0**	**1±0**	**1±0**	**1±0**	**0.96±0.07**	**1±0**	**1±0**	**1±0**	**1±0**
*Xeu* LMG 930	**0.95±0.06**	**1±0.01**	**0.9±0.1**	**0.98±0.02**	**0.83±0.2**	**0.99±0.01**	**0.98±0.02**	**0.99±0.01**	**0.94±0.04**
*Xeu* LMG 931	0.01±0.01	0±0	**0.96±0.04**	**0.98±0.02**	**0.85±0.16**	0.26±0.23	**0.99±0.01**	**0.98±0.02**	**0.98±0.03**
*Xeu* LMG 932	0.01±0.01	0.02±0.02	**1±0**	**1±0**	**1±0**	0.19±0.2	**1±0**	**1±0**	**1±0**
*Xeu* LMG 933	0.01±0	0±0	**1±0**	**1±0**	**1±0**	0.17±0.23	**1±0**	**1±0**	**1±0**
*Xeu* CPBF 404	0.02±0.01	0±0	**1±0**	**1±0**	**0.97±0.04**	0.01±0.01	**1±0**	**1±0**	**1±0**
*Xeu* CPBF 490	0±0	0±0	**1±0**	**0.99±0.03**	**0.99±0.01**	0±0	**1±0**	**1±0**	**1±0**
*Xeu* LMG 907	0±0	0±0	**1±0**	**1±0**	**1±0**	0±0	**1±0**	0.01±0.01	**1±0**
*Xv* LMG 911	0±0	0±0	0.06±0.06	0.02±0.01	0.03±0.04	0±0	0.02±0.02	**0.61±0.27**	0.01±0.01
*Xv* LMG 917	0±0	0±0	0.14±0.14	0.04±0.02	0.04±0.04	0±0	0.01±0.01	**0.93±0.12**	0.05±0.04
*Xv* LMG 919	0±0	0.02±0.02	0.07±0.09	0.05±0.03	0.03±0.04	0±0	0.02±0.02	0.01±0.01	0.03±0.04
*Xv* LMG 920	0.01±0	0±0	0.17±0.21	0.05±0.02	0.06±0.06	0.25±0.35	0.04±0.02	**1±0**	0.03±0.02
*Xv* LMG 923	0.02±0.01	0±0	0.09±0.12	0.03±0.02	0.04±0.03	0.06±0.08	0.04±0.03	**1±0**	0.05±0.06
*Xg* LMG 962	0.02±0.01	0.05±0.07	0.05±0.06	0.03±0.03	0.03±0.04	0.25±0.35	0.01±0.02	0.01±0.01	0.02±0.02
*Xg* NCPPB 4323	0.01±0.01	0±0	0.04±0.01	0.02±0.02	0.03±0.03	0.07±0.1	0.03±0.05	0.01±0.01	0.02±0.01
*Xg* NCPPB 4324	0±0	0±0	0.1±0.08	0.14±0.04	0.03±0.02	0.13±0.08	0.06±0.11	0.01±0.01	0.01±0.01
*Xp* NCPPB 4321	0.01±0.01	0±0	0.09±0.09	0.04±0.02	0.05±0.05	0.13±0.11	**1±0**	0.01±0.01	0.02±0.01
*Xp* NCPPB 4322	0.01±0.01	0.01±0.01	0.14±0.15	0.02±0.02	0.01±0.02	0±0	**1±0**	0±0.01	0.02±0.01

The displayed values refer to: average probability ± standard deviation.

Probabilities with an average value higher than 0.5 are highlighted in bold.

In general, the results confirmed the stability of markers XV6, XV7, XV8, XV11, XV12 and XV14 for most of the *Xeu* strains tested, since robust hybridization signals with high probability values were obtained (≥0.61), except for strain *Xeu* 913, which showed a low probability for marker XV8 (≤0.33). On the contrary, a different pattern was observed for strain *Xeu* 904, which showed weak dot signals sustained by low probability values for markers XV6, XV8, XV12 and XV14 (≤0.16). Interestingly, this strain, which is not clustered with the other *Xeu* as shown by the Neighbor-Joining tree (Fig. 4), has also an unusual placement in the phylogenetic trees obtained by Ah-You *et al.* [60], meaning that its identification as *Xeu* may not be accurate. Clearly, further studies with strain LMG 904 will be essential to provide further insight for the correct taxonomic positioning of this strain.

Concerning the markers XV4, XV5 and XV10, reliable hybridization and probability values (≥0.93), were obtained for only three strains, *Xeu* 922, *Xeu* 929 and *Xeu* 930, all isolated in the USA. On the other hand, and in addition to the *Xeu* strains some markers hybridized to a broader number of BSX strains, namely marker XV11 shown to hybridize strongly to the two *Xp* strains analyzed (*Xp* NCPPB4321 and 4322) with a probability value of 1±0 and marker XV12 with consistent hybridization signals recorded for four of the five *Xv* used (≥0.61).

To assess unspecific binding, the probes corresponding to each of the markers were also assayed with a collection of 12 non-BSX *Xanthomonas* and 23 non-*Xanthomonas*. The dot blot results strengthen the specificity of all the probes for BSX strains, as no hybridization signals were detected (See Fig. S4).

### Duplex PCR

Aiming to develop a time-efficient *Xeu* detection method using the molecular markers characterized in this study as an alternative to the dot blot and particularly suitable for plant material, a duplex PCR was optimized using a low size marker of 210 bp (XV7) and a larger marker of 631 bp (XV11). Both markers were chosen because they were shown to hybridize consistently to all the *Xeu* strains used in this study (Table 3). The duplex PCR was extensively validated using DNA from all strains listed in Table 2 and the results confirmed its efficiency for all the *Xeu* strains used (See Fig. S5). For the non-*Xeu* BSX, amplification was observed for some *Xv* and *Xg* strains, although with a significant loss of reaction efficiency. In agreement with the dot blot validation, a strong PCR product was obtained with marker XV11 for *Xp* strains. In relation to the non-BSX strains analyzed, no amplification was observed, with exception for *Xoo* 5047. Nonetheless, in this case the size of the three obtained amplicons suggests unspecific amplification. To estimate the detection threshold of the duplex PCR, which is particularly important to evaluate the effectiveness for the direct detection of *Xeu* in infected plant material, different concentrations of DNA and a diverse number of bacterial cells were used as templates (See Fig. S6). This assay allowed to detect as little as 2.5 pg of purified DNA and 10^2^ cells per mL of *Xeu* 905, using an increased initial PCR denaturation step of 10 min.

### Detection of *Xeu* in infected tomato and pepper plants

To assess the robustness of the selected markers for detection of *Xeu* directly from infected plant material, i.e. without previous isolation in culture, tomato and pepper plants were inoculated with different BSX (*Xeu* 905, *Xv* 919, *Xg* 962 and *Xp* 4321) and *Pst* DC3000, as a control for non-BSX infection. One and two weeks after inoculation single tomato and pepper plant leaves, with unnoticeable or minor disease symptoms, were collected and processed using a simple and fast procedure to produce a crude bacterial suspension to use directly in duplex PCR reaction and for inverted dot blot analyses.

**Figure 5:**
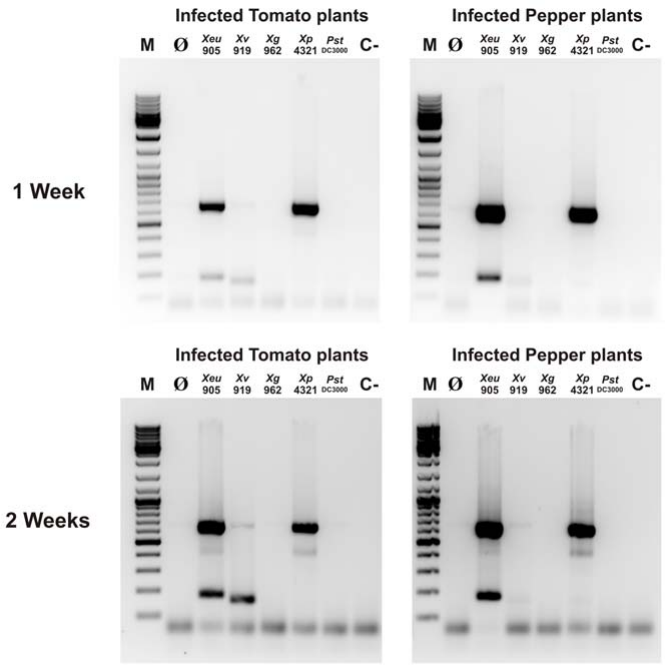
Detection of BSX in infected plant material using a duplex PCR (markers XV7 and XV11). Tomato and pepper plants inoculated with *Xeu* 905, *Xv* 919, *Xg* 962 and *Xp* 4321were processed after one and two weeks to obtain crude bacterial suspensions used as PCR templates. Plants inoculated with *Pst* DC3000 were used as controls. M – DNA marker (GeneRuler DNA Ladder Mix); Ø-Duplex PCR using distilled water as template; C- healthy tomato and pepper plants.

The duplex PCR results showed that infectious *Xeu* 905 was detected in both plants for both markers (XV7 and XV11) and whatever the experimental conditions (Fig. 5). Concerning the other BSX, the results confirmed the previous validation of markers XV7 and XV11 for these strains and supported the adequateness of using these markers to detect specifically *Xeu* in infected plant material (See Fig. S5). In fact, while only marker XV11 was amplified with plant material infected with *Xp* 4321, no specific amplification was observed with samples from plants inoculated with *Xv* 919 and *Xg* 962. The low size fragment obtained with tomato samples inoculated with *Xv* 919 after two weeks of inoculation is below the expected size for marker XV7 (212 bp), suggesting unspecific binding (Fig. 5). Lastly, and as expected, no amplification was obtained for plants sprayed with *Pst* DC3000, a tomato infectious pseudomonad.

**Figure 6:**
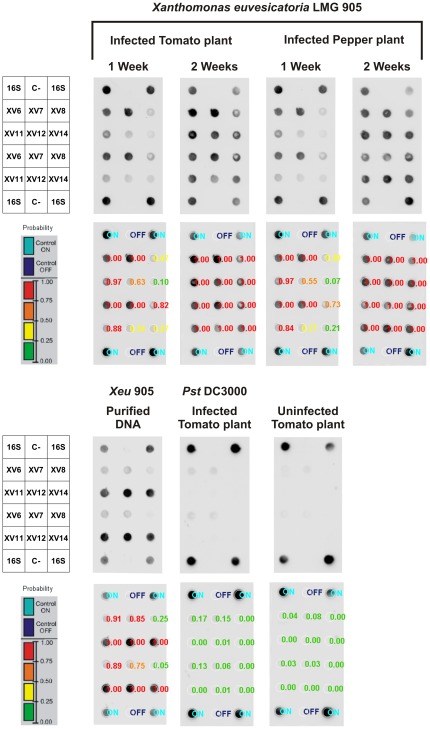
Detection of BSX in infected plant material using an inverted dot blot platform. Crude bacterial suspensions, obtained from tomato and pepper plants leaves after one and two weeks of infection with *Xeu* 905, were used as templates for PCR enrichment using the markers' primer pairs. PCR products corresponding to each plant were labeled with Digoxigenin and used as probes. Purified DNA from *Xeu* 905 was used as positive control. Negative controls consisted of tomato plants infected with *Pst* DC3000 for 2 weeks and uninfected plants. The raw ChemiDoc captures and processed images, using the automatic image analysis algorithm, are shown.

To improve diagnostics reliability and increase the detection consistency, an inverted dot blot platform, coupled with an automatic data analysis, was implemented using all the markers validated for detection of *Xeu* (XV6, XV7, XV8, XV11, XV12 and XV14). To increase the hybridization signal and before DIG labeling, the crude bacterial suspensions, obtained from the infected plant material and used as probes, were enriched by a heptaplex PCR using primer-pairs for the six markers (XV6, XV7, XV8, XV11, XV12 and XV14) together with a 16 S rRNA primer-pair, used as positive control. The results exhibited dot blot patterns indicative of *Xeu* 905 infection both in tomato and pepper plants, in contrast to tomato plants inoculated with *Pst* DC3000 which lead to negligible hybridization signals obtained for markers XV6 and XV7, i.e. with very low probability of being ON (≤0.17 and ≤0.15, respectively). Furthermore, despite the fact that probes corresponding to one week old infected plant samples did not show a clear hybridization with three markers (XV8, XV12 and XV14), the probes corresponding to two weeks old infected plants samples provided a consistent hybridization for all the markers and for tomato and pepper plants, easily recognized by the red color corresponding to high probability values ( = 1.00) (Fig. 6). The color gradient of the hybridization probability values is a helpful approach to immediately identify the likelihood of a dot being ON.

## Discussion

It is generally acknowledged that the early detection of BSX is the most effective measure to prevent bacterial spot disease dissemination. Presently, the reference diagnostic protocols carried out by the phytosanitary authorities rely on culture-based approaches, bacteria isolation in semi-selective media or serological detection methods [3]. However, these procedures are excessively time consuming, costly and laborious, which is a major drawback for a routine and extensive surveillance of these phytopathogens. In the advent of the genomic era, DNA-based methods are increasingly foreseen as rapid and accurate alternatives for the detection of these pathogens, surpassing the above mentioned limitations of the culture-based methods and allowing a high throughput screening [23]. Regardless of the breakthroughs in recent years [24], [61], including the reliable and rapid presumptive identification of some pathogens [25], [26], DNA-based detection methods are still lagging behind the long-established methods concerning their implementation by the regulators. Increasing confidence among the phytosanitary services, through the optimization of user friendly detection platforms and providing enhanced diagnostics resolution using novel and highly discriminatory molecular markers, is the impending challenge.

Over the last two decades numerous DNA-based approaches, mostly based on PCR techniques, have been proposed for the detection of numerous phytopathogens in general [62] and of BSX in particular. However, the primer-pairs proposed are not entirely specific for the target BSX, and a posterior restriction analysis to confirm the identity of the PCR fragments was required [29], [31], [32]. Recently, Moretti *et.al* (2009) [36] proposed the amplification of a promising 1.6 Kb *Xeu*-specific fragment, discovered through repetitive extragenic palindromic sequence-PCR (rep-PCR). Nevertheless, the BLAST analysis of the deposited sequence corresponding to this fragment (accession number FJ445513), revealed high similarity of 60% and 80% with *X. perforans* and *X. axonopodis* pv. *citri* str. 306, respectively. Concerning hybridization-based methods, specific DNA probes were developed targeting copper resistance genes in BSX strains, although these only allow the detection of Cu^r^ strains [28]. Kuflu *et al.* (1997) developed a dot blot platform for detection of *Xanthomonas axonopodis* pv. *vesicatoria* and *Xanthomonas vesicatoria* using a fragment obtained through genomic subtraction (KK1750), but the probe was not fully specific for BSX [35]. Furthermore, the PCR amplification of marker KK1750 was negative for some BSX strains [63].

The taxa-specific markers used in the above-mentioned studies, were either based on known functional genes, or discovered by experimental approaches namely with fingerprinting methods or subtractive hybridization. These two strategies to select DNA markers have strong limitations. In fact, while the use of functional genes to select DNA signatures demands a detailed knowledge of the target organisms biology and only allows to design a limited number of markers, the experimental screening of discriminatory genomic regions that might be used as detection markers, requires previous and extensive laboratorial validation, and does not provide relevant information regarding their genomic stability or intra-specific variability.

To tackle these limitations, an *in silico*-based DNA signature pipeline, based on the CUPID [41] and Insignia [42] databases, was employed. Although both resources have been developed to retrieve genus-, species- or strain-specific molecular markers, the outputs, i.e. number of specific proteins in CUPID and DNA signatures in Insignia, is very high, ranging from several dozens of specific proteins (149 for *Xeu*) to several hundreds of DNA signatures (15533 for *Xeu*), which is an unfeasible number of markers to validate. The idea to overlap the data from both databases, through the development of a C program, allowed to obtain a much more manageable set of markers. Indeed, the Insignia outputs were narrowed down to DNA signatures present in putative ORFs, obtained by CUPID. Furthermore, the follow-up BLAST analysis ensured the *in silico* specificity of the selected markers within the DNA databases. This is particularly important because the algorithms are not absolutely effective in determining taxa-specific regions. Actually, while gene banks (NCBI) are constantly updated with new DNA sequences, these are not regularly recalculated by CUPID and Insignia. The C program permitted to filter the initial dozens of putative specific regions to a discrete number of genomic regions with the discriminatory potential of a DNA-signature. Using the proposed *in silico* pipeline several specific regions were selected in order to design putative *Xeu*-specific DNA markers. No relevant BLAST (blastn) hits were obtained for most markers, excepting markers XV7 and XV11, which displayed significant similarity values with *X. axonopodis* pv. *citrumelo* F1 (Table 1). The analysis of the genome sequence of this strain revealed that it is very closely related to *Xeu* 85-10, however the two phytopathogens have no known matching hosts [57].

Aiming to understand how these markers became “unique” within *Xeu* and to evaluate their stability, the evolutionary history of these putative specific regions was examined. We hypothesized that a phylogenetic insight and/or a comparative genomic analysis of each *Xeu*-specific loci would provide valuable information to select the most promising markers, i.e. the markers shown to be evolutionary more distant from any other bacteria taxa. This would allow to pinpoint the bacteria taxa that should be primarily used for the experimental validation trials and ultimately the likelihood of these markers being present within all the members of the species *X. euvesicatoria*. Interestingly, the results showed that most of these putative markers were most likely obtained through horizontal gene transfer (HGT) events and had a phage-related origin. The low GC percentage [64], low values of CAI and normalized CAI [58] and the insertion events revealed through the synteny analysis are all indicative of a horizontal gene transfer origin for these markers. This hypothesis is strengthened by the presence of mobile genetic elements and other indicators of genome plasticity, namely phage related ORFs, IS elements, tRNAs, and enzymes involved in genomic rearrangements in the genomic vicinity of most of the markers.

After this comprehensive validation, ten primer-pairs were designed to amplify fragments ranging from 123 bp to 830 bp, within nine putatively discriminatory regions distributed throughout the *Xeu* chromosome. The PCR validation (Fig. 2) showed that six markers (XV6, XV7, XV8, XV11, XV12 and XV14) provided positive amplification with the eight tested *Xeu* strains, whatever their geographic origin and MLST profile (Fig. 4), suggesting the evolutionary stability of these markers within the species. The sequences mismatches observed for these markers across the different *Xeu* strains (see Table S1), is consistent with highly conserved genomic regions, contrary to what the HGT and phage related origin might evoke. Therefore, one might theorize that the genomic regions, within which these markers are located, were present in the *Xeu* common ancestor and their presumable genomic instability, as suggested by the comparative genomic analysis, has been lost due to inactivation of the mobilization-related features [65] leading to a vertical heritance of these loci within the *Xeu* species. On the other hand, markers XV4, XV5 and XV10, which were designed within the genomic regions located close to the origin of replication, only amplified with one of the tested *Xeu* strains and XV13 with none. These results suggest that these primers have a narrow-range and only target a subset of *Xeu* strains.

To broaden the specificity tests to a larger collection of BSX strains representative of a large geographic and genomic diversity (Table 2, Fig. 4), including several *Xv*, *Xg* and *Xp* strains, an hybridization-based validation, using a dot blot platform, was optimized. The data confirmed XV6, XV7, XV8, XV11, XV12 and XV14 as broad spectrum markers, specific for *Xeu* strains (Fig. 3). However, XV11 hybridized with the two tested *Xp* strains and XV12 provided additional signals with some of the *Xv* strains studied, revealing affinity to closely related BSX. It is important to emphasize that this noticeable hybridization to non-*Xeu* BSX strains, was not predicted by the previous *in silico* validation, most likely due to the limited genomic information available. Indeed only the BSX strain *Xeu* 85-10 had his chromosomal full sequence available. The draft genome sequences of *Xv*, *Xg* and *Xp*, that were recently made available [66], will certainly contribute to a more reliable prediction of specific regions for the different BSX species by CUPID and Insignia. A BLAST analysis carried out with the NCBI whole-genome shotgun contigs (wgs) database allowed to extend the *in silico* specificity tests to include these draft genomes. In accordance with the validation studies, marker XV11 presented relevant similarity with a sequence in the draft genome of *Xp* 91-118. Similarly, marker XV12 provided a significant BLAST hit with *Xv* ATCC 35937. No other relevant hits were obtained with exception of marker XV6, for which relevant similarity was observed with the draft genomes of *X. campestris* pv. *musacearum* ‘Kenyan’ and NCPPB 4381 and *X. campestris* pv. *vasculorum* NCPPB 702.

Regarding the markers XV4, XV5 and XV10, the hybridization profiles confirmed the PCR data and their occurrence in a restricted number of strains (*Xeu* 922, *Xeu* 929 and *Xeu* 930). Despite the lack of data to elucidate the genotype of these three strains, their common origin (USA) might suggest a shared genetic patrimony and justify their similar and exclusive behavior to these markers.

The dot blot specificity tests, carried out with a collection of non-BSX strains, confirmed the specificity of the markers, as no positive hybridization signals were recorded for any of the tested strains (see Fig. S4), underlining the adequateness of *in silico*-based approaches for high-quality DNA signature predictions. Similarly to PCR assays, no differences were observed in the performance of the small and larger DNA probes concerning hybridization efficiency or probe specificity.

Acknowledging the importance to develop a culture-independent detection method for *in planta* diagnostics of BSX disease a duplex PCR and an inverted dot blot approach were implemented.

For the duplex PCR, markers XV7 and XV11 were chosen because these were shown to be present in all the *Xeu* strains tested as demonstrated by the dot blot (Table 3) and duplex PCR (See Fig. S5) validation. Furthermore, the amplicon sizes of 179 bp and 631 bp, respectively, allow a clear discrimination between both markers. The validation assays, carried out with all the phytopathogens used in this work (Table 2), confirmed the adequateness of these two markers to identify presumptively *Xeu*, despite the faint amplification obtained for some non-*Xeu* BSX, namely *Xv* and *Xg* strains or the amplification of marker XV11 for *Xp* strains. In fact, when using tomato and pepper plants inoculated with different BSX and a straightforward bacterial DNA extraction protocol as described above, the duplex PCR trustworthily detected *Xeu* as early as 1 week after infection of both plant species (Fig. 5). Therefore, this procedure can be a helpful alternative to the presently used methods of diagnostic for an immediate and assertive diagnostic of *Xeu* in symptomless plants, without the need for sample enrichment or isolation in semi-selective media [3]. Due to the possibility that non-*Xeu* xanthomonads may result in uncertainties concerning the identity of the BSX species detected by the presumptive duplex PCR assay, a complementary hybridization-based assay, using six markers was optimized to detect *Xeu* in infected plant material. This inverted dot blot platform allowed the detection of *Xeu* in crude bacterial suspensions obtained from infected tomato and pepper plants and whatever the tested conditions (Fig. 6). This macroarray-based detection assay, that can be used as a confirmatory assay for the duplex PCR, can easily be expanded to include several other DNA markers in order to allow the reliable detection of a vast range of phytopathogens and overcome the main disadvantages of microarray technology for routine detection [23], [67], particularly their cost and data analysis complexity.

Overall, in this work we propose an efficient DNA signature discovery pipeline using Insignia and CUPID, capable of providing a consistent number of DNA signatures for a target taxon. In addition, we demonstrated how a comprehensive evolutionary validation of markers using comparative genomics analyses, might provide valuable information about markers' specificity and stability, i.e. the likelihood of the markers to be present within all the members of the target taxon, which can never be achieved by experimental validation alone. Finally, we developed a duplex PCR and a dot blot platform as two efficient culture-independent methods for detection of *Xeu* in infected plant material. While these two cost-efficient techniques were shown to be effective for *Xeu* detection, it is important to emphasize that the proposed specific DNA regions can be easily used as targets to design new primers or probes suitable for alternative detection techniques, namely Real-Time PCR. Ultimately, we expect that this work might constitute a solid ground to improve new phytodiagnostics methods and to introduce broad, simple, reliable, and cost-efficient protocols that might be easily welcomed by the certified phytosanitary services as advantageous alternatives or extensions to the currently used methods.

## Supporting Information

Figure S1 **Whole genome syntenic dotplots and comparative synteny maps of** ***Xeu*** **85-10 and** ***Xaci*** **306.** The location of each marker is indicated by an orange arrow. The pink blocks shown in the syntenic map represent syntenic genomic regions between both genomes and the gaps indicate non-syntenic regions.
(TIF)

Figure S2 **Whole genome syntenic dotplots and comparative synteny maps of** ***Xeu*** **85-10 and** ***Xcc*** **33913.** The location of each marker is indicated by an orange arrow. The pink blocks shown in the syntenic map represent syntenic genomic regions between both genomes and the gaps indicate non-syntenic regions.
(TIF)

Figure S3 **Whole genome syntenic dotplots and comparative synteny maps of** ***Xeu*** **85-10 and** ***Xoo*** **311018.** The location of each marker is indicated by an orange arrow. The pink blocks shown in the syntenic map represent syntenic genomic regions between both genomes and the gaps indicate non-syntenic regions.
(TIF)

Figure S4 **Dot blot specificity validation.** Nine digoxigenin-labeled probes corresponding to nine markers were tested for specificity with 12 non-BSX *Xanthomonas* and 23 non-*Xanthomonas*, including the phylogenetically closely related *Sm* 958 and *Xllf* 17159. C+ refers to the positive control prepared with *Xeu* 905 genomic DNA as template.
(TIF)

Figure S5 **Duplex PCR validation.** A duplex PCR, targeting markers XV7 and XV11, was tested for specificity using all the bacteria listed in Table 2, which included several BSX, non-BSX *Xanthomonas* and other phytopathogenic bacteria. M – DNA marker (GeneRuler DNA Ladder Mix); C+ refers to the positive control obtained with *Xeu* 905 genomic DNA as template; C- negative control (sterile distilled water).
(TIF)

Figure S6 **Duplex PCR detection limits.** The duplex PCR resolution was assessed using purified DNA from *Xeu* 905, *Xeu* 905 cells and plant material spiked with *Xeu* 905 cells. M – DNA marker (GeneRuler DNA Ladder Mix); C- negative control (sterile distilled water).
(TIF)

Table S1 **SNPs located in the markers.** Multiple alignments of the markers sequences (accession numbers HQ316640 to HQ316699), obtained from the different *Xeu* used in this study, allowed to identify SNPs (yellow boxes). The amplicons obtained with *Xp* strains (4321 and 4322) using marker XV11 were also sequenced and analyzed for sequence differences (shown in blue) and SNPs (shown in yellow). No SNPs were observed for markers XV4, XV5 and XV10, with the assayed strains.
(PDF)
