# Supplementary figures and images for: Evolutionary and Experimental Assessment of Novel Markers for Detection of Xanthomonas euvesicatoria in Plant Samples
Source: PLoS One. 2012 May 24;7(5):e37836. doi: 10.1371/journal.pone.0037836 (PMC3359998; doi:10.1371/journal.pone.0037836)

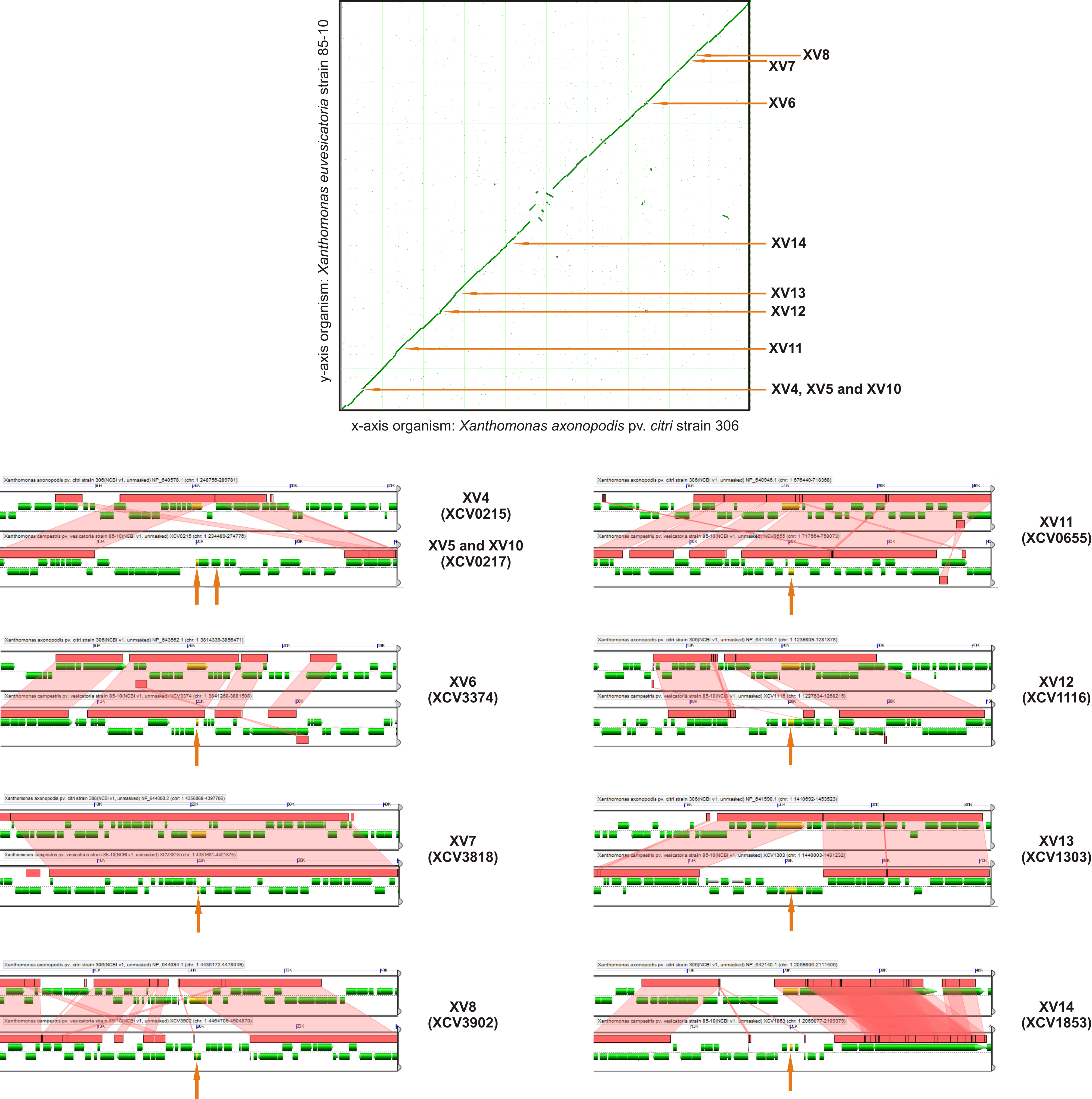

Supplement: Figure S1 — Whole genome syntenic dotplots and comparative synteny maps of Xeu 85-10 and Xaci 306. The location of each marker is indicated by an orange arrow. The pink blocks shown in the syntenic map represent syntenic genomic regions between both genomes and the gaps indicate non-syntenic regions. (TIF) [file pone.0037836.s001.tif]

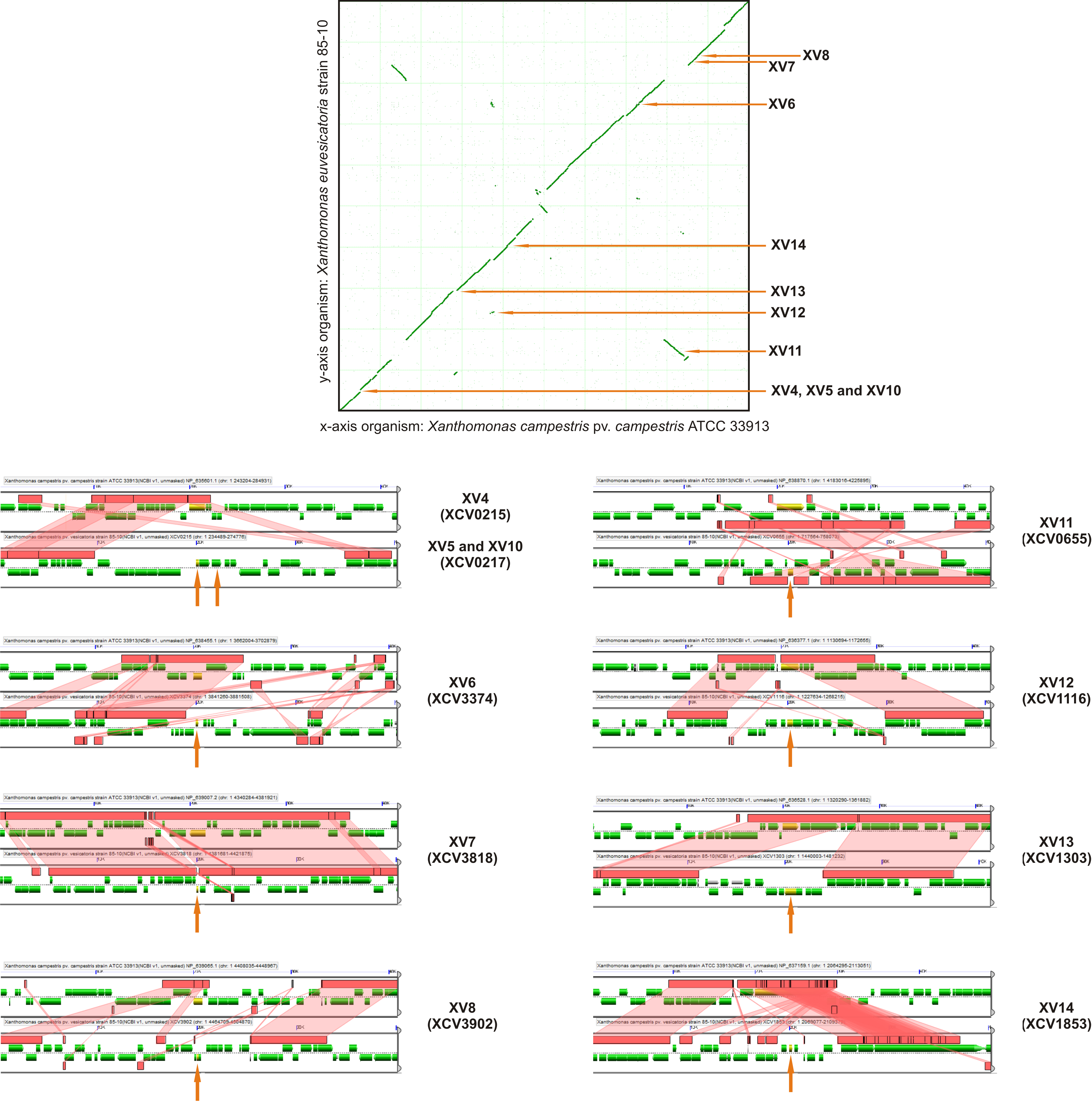

Supplement: Figure S2 — Whole genome syntenic dotplots and comparative synteny maps of Xeu 85-10 and Xcc 33913. The location of each marker is indicated by an orange arrow. The pink blocks shown in the syntenic map represent syntenic genomic regions between both genomes and the gaps indicate non-syntenic regions. (TIF) [file pone.0037836.s002.tif]

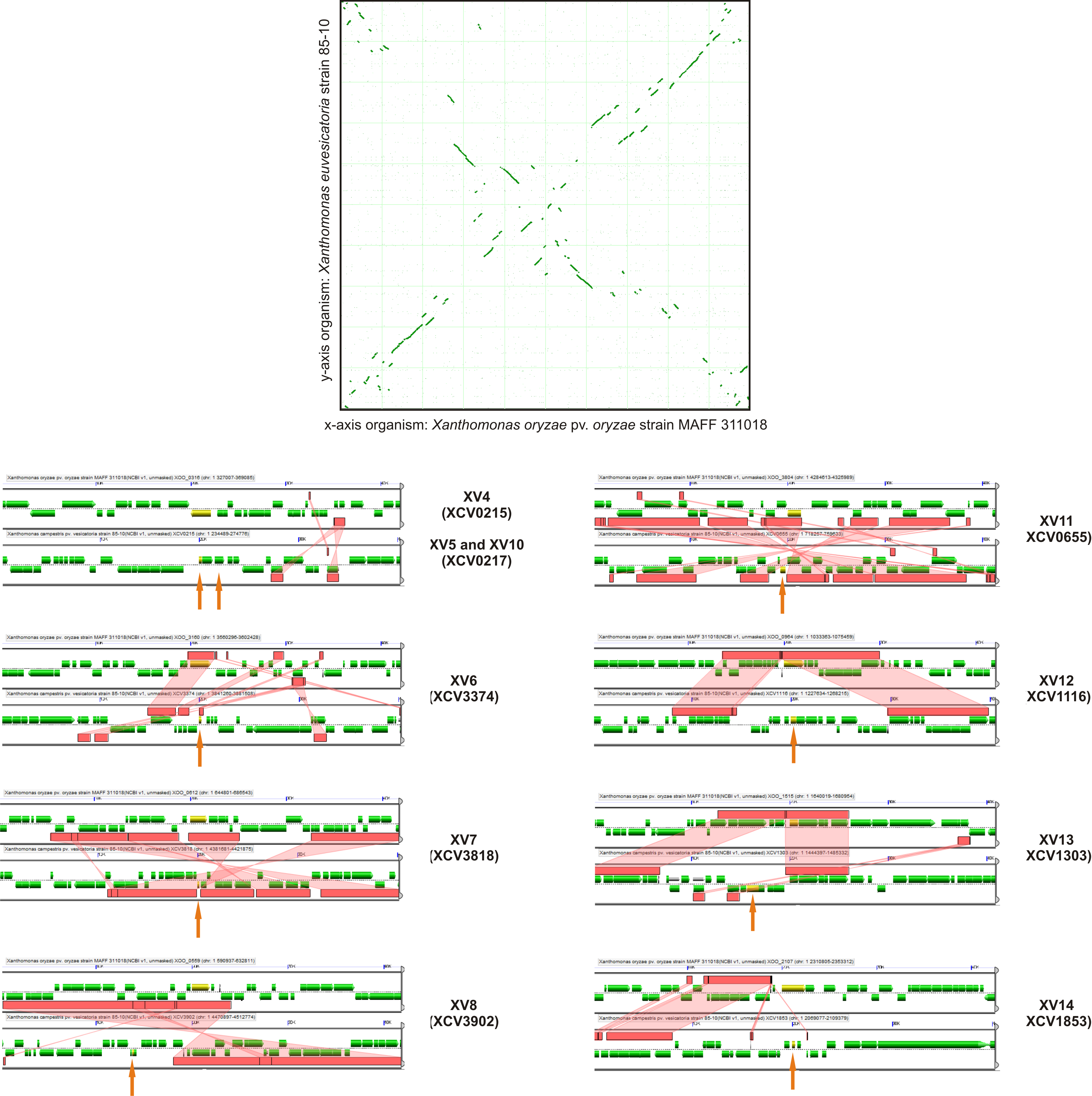

Supplement: Figure S3 — Whole genome syntenic dotplots and comparative synteny maps of Xeu 85-10 and Xoo 311018. The location of each marker is indicated by an orange arrow. The pink blocks shown in the syntenic map represent syntenic genomic regions between both genomes and the gaps indicate non-syntenic regions. (TIF) [file pone.0037836.s003.tif]

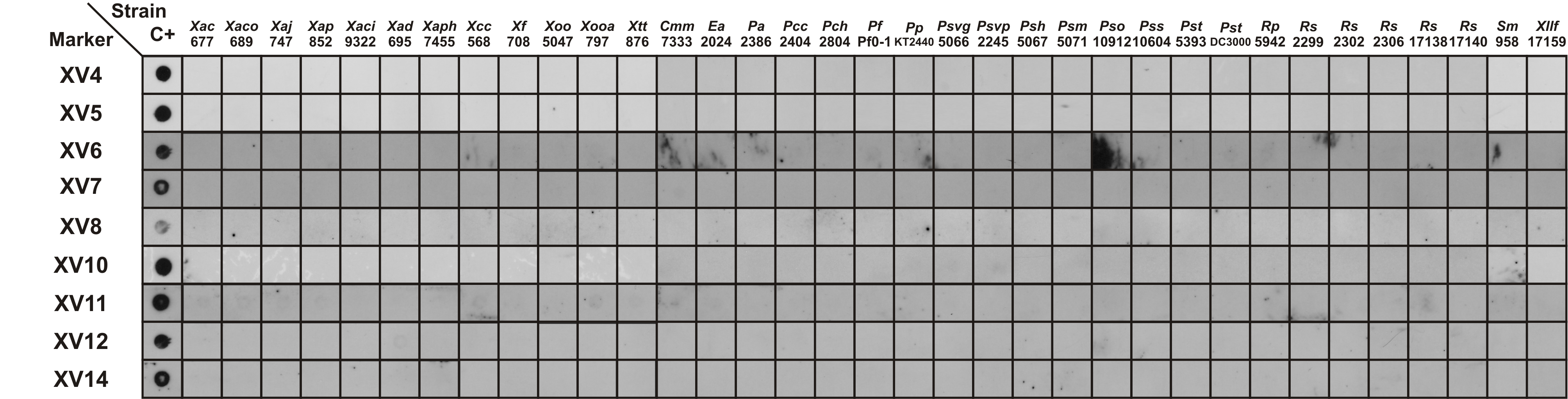

Supplement: Figure S4 — Dot blot specificity validation. Nine digoxigenin-labeled probes corresponding to nine markers were tested for specificity with 12 non-BSX Xanthomonas and 23 non-Xanthomonas, including the phylogenetically closely related Sm 958 and Xllf 17159. C+ refers to the positive control prepared with Xeu 905 genomic DNA as template. (TIF) [file pone.0037836.s004.tif]

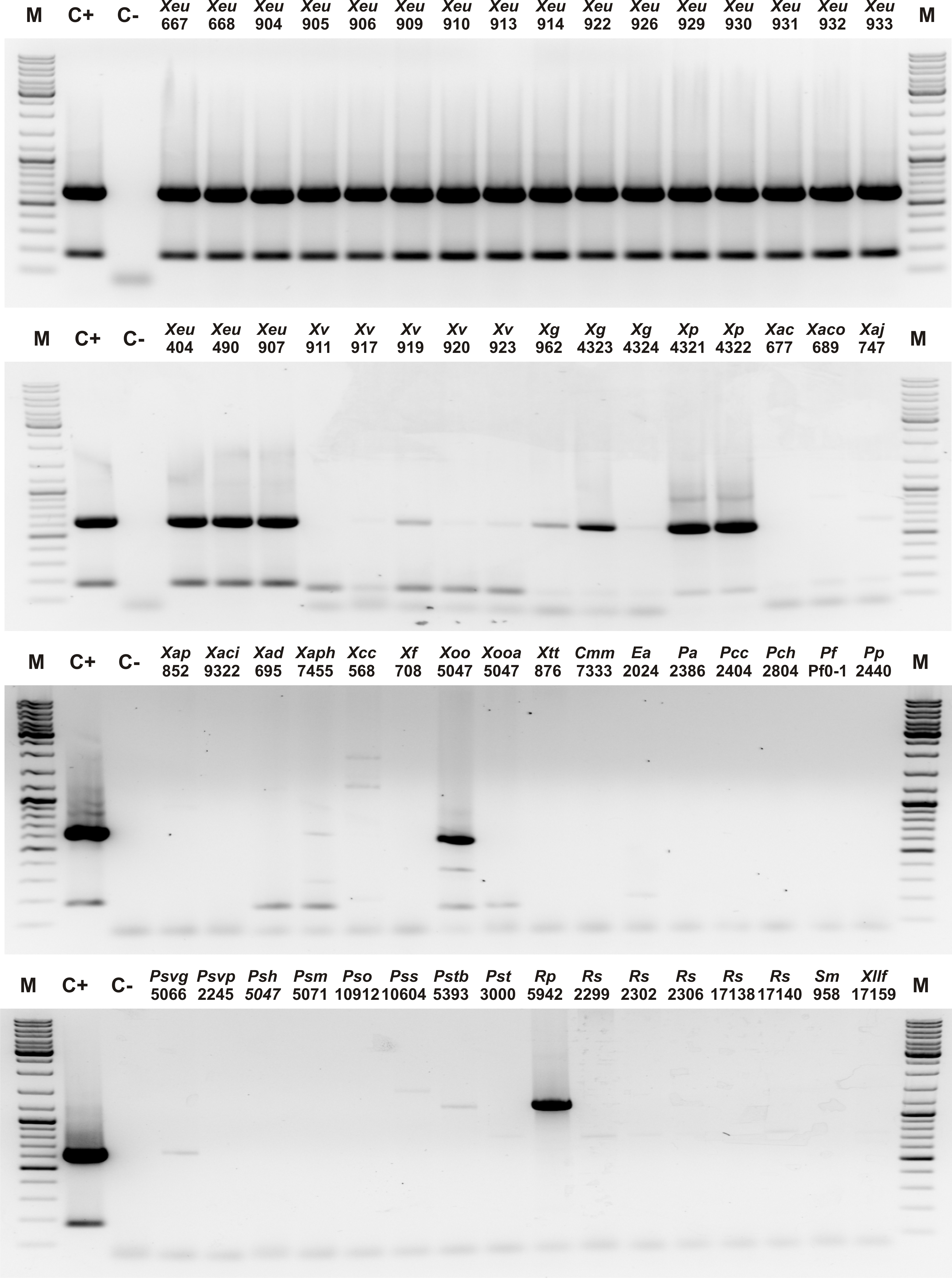

Supplement: Figure S5 — Duplex PCR validation. A duplex PCR, targeting markers XV7 and XV11, was tested for specificity using all the bacteria listed in Table 2, which included several BSX, non-BSX Xanthomonas and other phytopathogenic bacteria. M – DNA marker (GeneRuler DNA Ladder Mix); C+ refers to the positive control obtained with Xeu 905 genomic DNA as template; C- negative control (sterile distilled water). (TIF) [file pone.0037836.s005.tif]

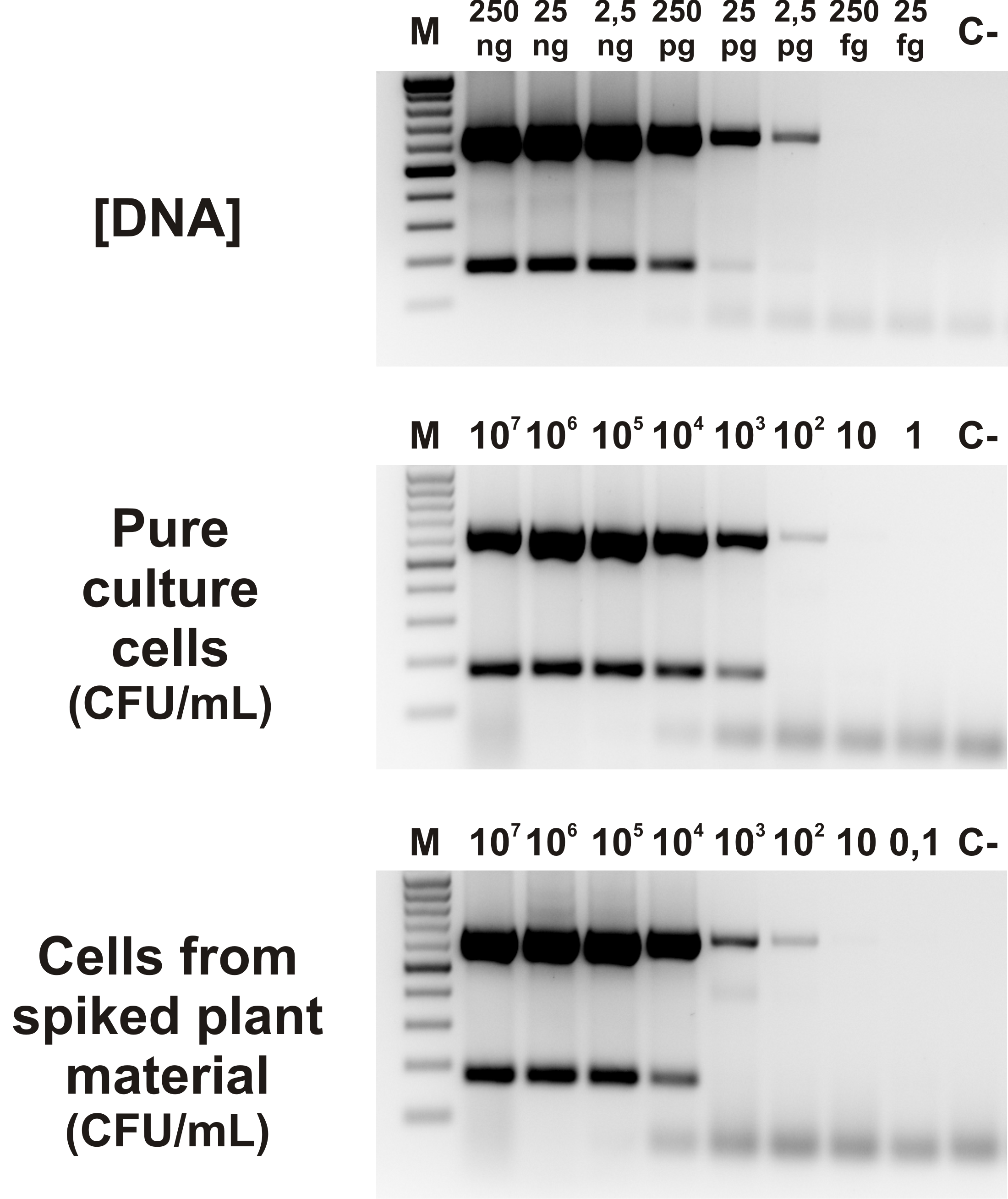

Supplement: Figure S6 — Duplex PCR detection limits. The duplex PCR resolution was assessed using purified DNA from Xeu 905, Xeu 905 cells and plant material spiked with Xeu 905 cells. M – DNA marker (GeneRuler DNA Ladder Mix); C- negative control (sterile distilled water). (TIF) [file pone.0037836.s006.tif]
